# Recommendations for collecting and analysing migration-related determinants in public health research

**DOI:** 10.25646/11144

**Published:** 2023-03-21

**Authors:** Katja Kajikhina, Carmen Koschollek, Navina Sarma, Marleen Bug, Annelene Wengler, Kayvan Bozorgmehr, Oliver Razum, Theda Borde, Liane Schenk, Ruth Zimmermann, Claudia Hövener

**Affiliations:** 1 Robert Koch Institute, Berlin Department of Epidemiology and Health Monitoring; 2 Robert Koch Institute, Berlin Department of Infectious Disease Epidemiology; 3 AG2 Population Medicine and Health Services Research, School of Public Health, University of Bielefeld; Section Health Equity Studies & Migration, Universitätsklinikum Heidelberg; 4 AG3 Epidemiologie & International Public Health, School of Public Health, University of Bielefeld; 5 Alice Salomon Hochschule Berlin; 6 Institute of Medical Sociology and Rehabilitation Science, Charité Berlin

**Keywords:** MIGRATION BACKGROUND, MINIMUM INDICATORS, SOCIAL DETERMINANTS, DISCRIMINATION, EXPLANATORY MECHANISM

## Abstract

**Background:**

According to the definition of the German Federal Statistical Office, about every fourth person living in Germany has a so-called migration background (MB), i.e., the person or at least one of their parents was born without German citizenship. However, MB has been defined differently in many studies. Also, the MB summarises people in different living situations, making differentiated analysis in health science more difficult. This article formulates recommendations for the collection and analysis of migration-related, as well as social and structural, determinants of health.

**Indicators for capturing relevant determinants of health:**

As part of the Improving Health Monitoring in Migrant Populations project (IMIRA), the previous approaches to operationalise and measure migration-related determinants were revised based on literature research and exchange formats, such as workshops, meetings, congress contributions, etc. Instead of MB, the country of birth of the respondents and their parents, duration of residence, citizenship(s), residence status, and German language proficiency should be recorded as minimum indicators and analysed as individual variables. Further social and structural determinants, such as socioeconomic position, working and housing conditions, or self-reported discrimination, should be included.

**Conclusions:**

In order to describe health inequalities and to specifically identify the needs of people with a history of migration, a mutual and differentiated consideration of migration-related and social determinants of health is essential.

## 1. Background

### 1.1 Migration in Germany

Germany is a country of immigration – the society in this country looks back on a long history of different migration movements. Examples include labour migration as part of the recruitment agreements from 1955 to 1973 or the regulations on the European Union (EU) Freedom of Movement Act from the 2000s. Refugee migration also plays a role, e.g., as a result of the military conflicts in the territory of the former Yugoslavia from 1991 to 2001 or in recent years as a result of the ongoing wars in, e.g., Syria and Ukraine [1, 2]. Added to this, people also immigrate for family reunification or for the purpose of training and studying. Therefore, migration is dynamic, and the people who migrate are a heterogeneous group of people with diverse biographies and living situations. Nevertheless, summarising categories are used for official statistics and in health science, and analysis is carried out using aggregated variables, such as ‘migration background’ (Table 1).

### 1.2 Criticism of the concept of ‘migration background’

The category ‘migration background’ is criticised due to different (Table 1) or partly unclear operationalisations, both in research and by migrant self-organisations or non-governmental organisations [7–9]. The term is often used without a clear definition and is sometimes confused with categories, such as country of birth, citizenship, or language proficiency, or these indicators are mixed up to form the variable. For example, in the German Health Survey for Children and Adolescents (KiGGS), the language spoken at home was used to define the ‘migration background’ if information on the country of birth and citizenship of the parents was missing [6] (Table 1). Another reason is the large number of required survey questions. In the micro-census, for example, 19 items were collected to derive the category of ‘migration background’ [10]. In addition, the concept is only used in the German-speaking parts of the world. The logic of foreign attribution is also criticised, which means that ‘migration background’is a concept that is primarily used by the majority of the society to describe certain groups of people but is usually rejected as a self-designation [11]. With ‘migration background’, a large number of different biographies and living situations are summarised into one variable. At the same time, the association of relevant determinants with health cannot be differentially modelled using this variable, such as racism in the form of experiences of discrimination or structural exclusions in access to social resources, such as housing, work, education, or health [8]. People can also experience racial discrimination even though they do not have a statistically defined migration background. Therefore, the category is not suitable for analysing and describing the diversity and inequality in today’s society.

**Info box 1**

**History of migration, migration background – what terms do we use to describe what?**

People with a migration background or history of migration, immigrants and their (direct) descendants, people with an international history – various terms have been used in recent years to speak about migration and about people living in Germany. In this article, we use the term ‘people with a history of migration’ to refer to people who have immigrated themselves or whose parents have immigrated; however, this term is not intended to replace the statistical category of ‘migration background’.

The concept of ‘migration background’ has been increasingly criticised for multiple reasons, for example, by migrant self-organisations or by the Federal Expert Commission on the Framework Conditions for Integration Capability [15]. Therefore, we suggest that the concept should no longer be applied. On the one hand, the ‘migration background’ is often operationalised in studies in the health sciences differently than in the official statistics. Studies often conflate country of birth and current citizenship [5, 6, 35], whereas the definition of the Federal Statistical Office refers to one’s own and/or parental citizenship at birth [4]. In the general public, the term is often applied without a clear definition and serves to describe people who are German but are supposedly perceived as ‘not from here’. Since its introduction, the term has also experienced a development towards a stigmatising attribution to others [8] and is now mostly rejected as a self-description.

**History of migration, migration background – what terms do we use to describe what?**

In contrast, the term ‘people with a history of migration’ is often used as a self-description of people who immigrated themselves or whose families have a biographical reference to migration or flight. Again, this term describes a very heterogeneous group of people. Therefore, rather than using aggregate categories such as ‘migration background’ or ‘history of migration’, we recommend analyzing relevant migration-related single indicators combined with other social determinants of health, depending on the particular research question for a differentiated analysis of migration and health. This approach is essential for making differentiated conclusions about factors and explanatory mechanisms of health inequalities.

At the same time, the term has developed from a statistical to a social category [12, 13] and to a category that tends to be exclusive and attributed to others. In the public media and in everyday understanding, it is often used to describe non-*white*, racialised groups of people who do not necessarily have a ‘migration background’ in the officially defined sense. The term is then understood to mean people who are not considered ‘typically German’, who are supposedly foreign and do not belong. The term is therefore accompanied by a clear discrepancy between its use in society and the media and its application in official statistics [14].

In 2021, the Federal Expert Commission on the Framework Conditions for Integration Capability (Expert Commission on Integration Capability) recommended shifting away from the ‘migration background’ as a statistical category [15]. Instead, this category is recommended to be replaced by ‘immigrants and their (direct) descendants’. This category only includes people who either immigrated themselves or whose parents both have their own history of immigration (since 1950). Additionally, the current citizenship of the respondents should be recorded. The improved comparability, also in the international context, with concepts such as ‘migrants’ or ‘foreign-born’ [16, 17], as well as the simplified data collection in quantitative surveys, are some of the arguments with regard to the improved methodological applicability of the new category [15]. Nonetheless, this new category also sums up (groups of) people in different living situations; the diversity within the (groups of) people categorised this way is still not taken into account, and possible health-related determinants would not be shown if this analysis only take this new category into account.

### 1.3 Migration and health

In order to examine the health situation of people who have their own or a family history of migration (Info box 1), it is essential to do this in connection with the social determinants that shape their lives and influence their health. Public health research in the fields of migration, racism, and health should differentially record and analyse the following factors and their interactions: aspects of the history of migration or flight, experiences of discrimination and racism, and structural and living environment-related factors, such as social position, social environment, working conditions or income, and housing situation.

Over the course of the coronavirus disease 2019 (COVID-19) pandemic, it became clear that there were augmented outbreaks, e.g., in communal accommodations or in certain industrial sectors, such as meat processing [18–20]. People with a history of migration were more often affected; however, infections did not occur due to the history of migration itself. Rather, cramped living conditions, which are associated with an increased risk of infection, or working conditions with increased exposure to SARS-CoV-2 (severe acute respiratory syndrome coronavirus type 2) due to limited options for protection against infection and barriers in accessing multilingual information, appropriate prevention, and medical care, were decisive [20]. International research, as well as research from Germany, indicates more frequent severe disease progressions, more frequent hospitalisations, increased need for intensive care, and increased mortality rates among ethnic minorities or people with a history of migration [21–26]. Analysis from Berlin suggested increased infection rates in districts with a higher proportion of people with a history of migration, who are also more socially and economically disadvantaged [27]. Data from the Organization for Economic Co-operation and Development (OECD) also show that people with a history of migration are more likely to be affected by poverty [28]. Besides COVID-19, other chronic (non-communicable) diseases are associated with poverty, an increased risk of poverty, precarious working and living conditions, and a lower socioeconomic position [29–34]. These aspects should be considered as relevant explanatory contexts because health risks and inequality cannot be explained solely by the (supposed) history of migration.

In contrast, the term ‘people with a history of migration’ is often used as a self-description of people who immigrated themselves or whose families have a biographical reference to migration or flight. Again, this term describes a very heterogeneous group of people. Therefore, rather than using aggregate categories such as ‘migration background’ or ‘history of migration’, we recommend analyzing relevant migration-related single indicators combined with other social determinants of health, depending on the particular research question for a differentiated analysis of migration and health. This approach is essential for making differentiated conclusions about factors and explanatory mechanisms of health inequalities.

### 1.4 Data analyses on migration and health

Instead of a lump-sum considering the ‘migration background’ as a differentiating characteristic or as a statistical control variable, research questions with a reasonable content should form the basis of migration-related analyses. The focus must first be put on the question of which explanatory mechanisms, contexts, exposures, and risks are to be examined and which factors and interactions are relevant for the health outcome under consideration, instead of just focusing on supposed characteristics, such as ‘migration background’ [36].

Against this background, the analysis of social inclusion and exclusion mechanisms, their interactions, discrimination, privileges, and access paths to social and health resources is absolutely necessary in order to better understand health conditions and health opportunities. Thus, the collection and analysis of data considering the social situation, in particular, indicators of the socio-economic position, the working and housing situation, residence status, access restrictions to standard health care, and other social resources, such as education or access to healthy living environments, are essential for differentiated analyses and appropriate explanatory models. Furthermore, indicators such as the subjective social status or subjective social mobility in connection with one’s own experience of migration are recommended [37] to analyse factors of health inequality. On the other hand, analyses based solely on the category of ‘migration background’ are not expedient. Besides, such simplified analyses (re)produce differences and thus also exclusions.

So far, the operationalisation of the category of ‘migration background’ in health research studies at the Robert Koch Institute (RKI), and often in other institutions, has been based on the recommended minimum set of indicators for recording the migration status, according to Schenk et al. [35]. The aim of this article is to provide updated recommendations for collecting migration-related indicators but also for the collection of selected social and structural determinants of health.

## 2. Indicators for data collection on migration-related and further relevant social determinants of health

**Figure 1.**
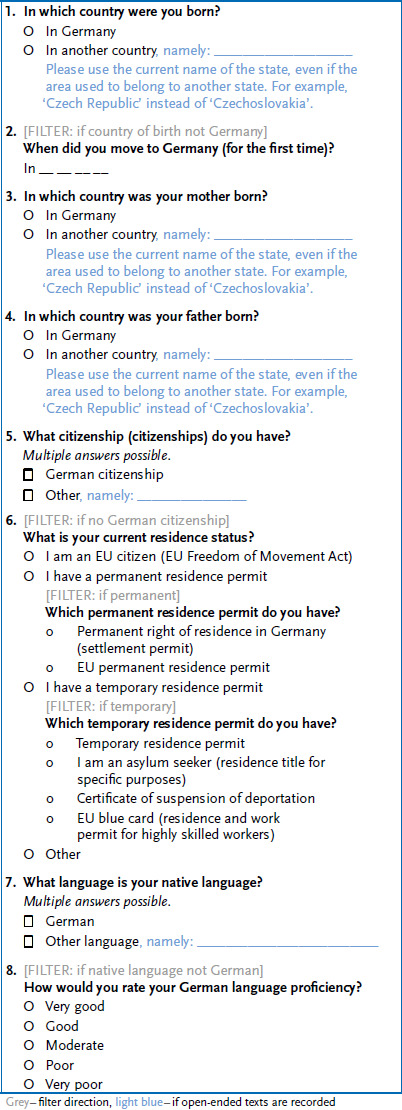
Operationalisation of the minimal indicators to record migration-related determinants Source: own depiction

Based on the existing minimum set of indicators [35] and considering the current state of the discussion, the following recommendations for minimum and additional indicators for surveying migration-related determinants were developed and are described below. In addition, we would like to propose the consideration of other social and structural factors in the analysis of the health status of people. The recommendations evolved as part of a joint reflection process based on discussions during different event formats like workshops, literature research, reviews, and cognitive pre-tests, as well as feasibility studies and interview surveys, as part of the Improving Health Monitoring in Migrant Populations project (IMIRA, started in 2016) [38–43]. The recommendations for the proposed indicators are also consistent with the recommendations of the Regional Office for Europe of the World Health Organization (WHO EURO): WHO EURO proposes recording the same indicators as ‘core’ or ‘recommended indicators’ [44]. The recommendations facilitate the connectivity to international research. They also include the renunciation from the concept of ‘migration background’ in health and social science surveys but also in the analysis of existing data sets. Rather, it is recommended to use relevant single indicators from the minimum set of indicators, depending on the research question. It is important to consider their interactions with the additional indicators presented below, considering important social and structural factors that are relevant to health (Table 2). Figure 1 shows our proposed operationalisation of the minimum indicators for the data collection of migration-related determinants.

### 2.1 Minimum indicators

We recommend recording the following minimum indicators in health research surveys and using them as single indicators in data analysis. This is also implemented in the ‘German Health Update (GEDA’ surveys of the RKI from 2020 onwards. Here, we merely refrain from collecting data on the self-assessed German language proficiency due to the data collection mode by telephone and in the German language only. The WHO Regional Office for Europe also recommends the regular collection of almost all of the indicators presented below in national public health monitoring [44].

#### Country of birth

First of all, we propose recording the country of birth. This can be recorded as Germany or being born in another country; the other country could also be captured as open-ended text. Using the variable country of birth, the experience of migration of a study participant can be depicted. Also, this indicator has international connectivity, as it is possible to compare people who migrated to those who did not migrate (‘migrants’ vs. ‘non-migrants’). Furthermore, recording the country of birth is beneficial for methodological analyses, e.g., to evaluate the sample composition and possible systematic bias in comparison to official population statistics, like the microcensus. If countries of birth are to be recorded with open-ended text, the summarising categorisation of the countries for the purpose of data analyses should follow official specifications, e.g., the categorisations of the United Nations (UN) [45], the World Health Organization (WHO) [46–51], or the Federal Statistical Office [52]. The recording of the country of birth also meets the recommendation of the Expert Commission on Integration Capability (see above). As a limitation, it is important to mention that country of birth is not necessarily equivalent to country of origin [44] as recorded in HIV surveillance [53]. Depending on peoples’ biography, ‘country of origin’ might be understood as their country of birth but also as the country in which most of their life has been spend or the country in which people had been living during the last years.

#### Countries of birth of the parents

We recommend recording the countries of birth of the parents following the same procedure. Using the countries of birth of parents, the category of ‘immigrants and their (direct) descendants’, proposed by the Expert Commission on Integration Capability, can be depicted; however, using this category in health research analyses also has limitations. In particular, this category is useful for comparisons with official population statistics to assess the quality of the sample composition.

#### Year of immigration

Using the year of immigration, the duration of residence can be depicted for persons being born abroad, which can be useful in the analyses of the conversion of disease risks over time [54]. Furthermore, a shorter duration of residence can be accompanied with barriers in accessing the German health system due to lack of information or legal restrictions [44]. For refugees, a shorter duration of residence is also accompanied with restricted access to health care according to §§4 and 6 of the Asylum Seekers Benefits Act [55, 56]. Also, for some groups of migrant workers (e.g., employees in harvesting, food processing, or construction industry within the framework of regulations on the EU Freedom of Movement Act), some restrictions on accessing regular health care exist, e.g., due to lack of social or health insurance or limited access to (multilingual) health information, which are often combined with higher health risks at work [41, 57, 58].

#### Citizenship

Furthermore, we recommend recording the citizenship, including possible dual (or multiple) citizenships, as German citizenship is linked to some relevant health privileges, e.g., accessing health care, working, and housing markets, as well as the social security system in Germany [44, 59]. For people with the citizenship of an EU country, certain access and mobility privileges exist, e.g. due to the regulations on the EU Freedom of Movement Act, compared to people who have neither German citizenship nor the citizenship of another EU country. Hence, citizenship, especially in combination with residence status, might be considered as a relevant indicator for health inequalities, determining access to health care [59–61]. Furthermore, this variable can give information about access to social and health-related resources, as well as about the health status of the population with and without German citizenship [26]. Also, this variable allows comparisons to official population statistics to assess the quality of the sample composition; however, as the citizenship is a changeable characteristic, it is recommended for longitudinal surveys to regularly update this information. If citizenship is recorded as open-ended text, categorisations should follow official specifications (see above, country of birth).

#### Current residence status

For study participants without German citizenship, we additionally recommend asking for the current residence status. For people with temporary or insecure residence status, differences in psychological strain and stressful experiences, as well as in terms of access to health care, are to be considered, which might further impact health outcomes [62, 63]. Since the residence status is a changeable characteristic, information on this is only valid for the survey time.

#### Self-assessed German language proficiency

Self-assessed German language proficiency can be recorded via two items. First, the native language is recorded. Here, the answer categories ‘German’ and ‘another language’ should be offered or the other language should be recorded via open-ended text. We recommend offering multiple answer options. Those not indicating German as their native language should then be asked to rate their German language proficiency on a five-point scale ranging from ‘very good’ to ‘very poor’. German language proficiency is an important indicator to analyse research questions concerning the access to health information and to health care, since both is accessible mainly in German language in Germany. Recorded as open-ended texts, native languages can give hints in which languages health information should be offered besides German. As a limitation, it should be noted that this indicator does not differentiate between spoken and written language proficiency, which might be important for the acquisition and understanding of health information, as well as in direct communication with health professionals. As the self-assessed German language proficiency might change over time, we recommend updating this information regularly for longitudinal studies.

### 2.2 Additional social and migration-related determinants

Next to the minimum indicators to record migration-related determinants described above, we recommend recording some additional indicators to consider in the analyses on the health status of people with a history of migration. These are indicators, with the exception of the reasons for migration, that are relevant to the whole population; however, for the health of people with a history of migration, these indicators are of particular importance as they present relevant aspects in (post) migration biographies. The selection of these indicators is based on the results of a systematic review and other research in the context of the IMIRA project [39, 42].

#### Reasons for migration

For people with their own experience of migration, it can be useful to ask for their reasons for migration. Voluntarily leaving the country of birth might have different impacts on health compared to forced migration, e.g., due to conflicts or war. Access to regular health care is subject to restrictions for refugees during their first time, after arrival, or permanently, depending on their residence status. Also, the experience of flight or expulsion can have long-term impacts on health. In surveys, it should always be possible to indicate multiple reasons for migration, as in practice, there might be mixed motives more often than a clear singular reason. Reasons for migration can be captured with the question: ‘What were the main reasons for you moving to Germany?’. Offering the possibility of multiple answers, possible answer categories are e.g., ‘to work’, ‘to study or for training and education’, and other answer categories reffereing to, e.g., family reunification or different reasons for flight (war, political persecution).

#### Self-reported discrimination

Special attention needs to be paid to capturing subjectively perceived or self-reported discrimination, as discrimination might have a number of direct or indirect associations towards the general, physical, and mental health of those affected. It is vital to record everyday experiences of discrimination, as well as discrimination in public institutions and health care facilities. Multiple possible reasons for experiencing discrimination should be offered. Moreover, experiences of discrimination might impact trust in health care facilities; however, trust in these institutions is essential for access to health care [42, 64, 65]. Recommendations for the operationalisation of self-reported discrimination are described elsewhere [41, 42].

#### Social support

As a psychosocial resource, social support has an immense impact on the health of people. Social support can facilitate coping with stress and can positively affect peoples’ overall physical and mental well-being [66–68]. Social support can be recorded using the Oslo Social Support Scale (OSSS-3) [69].

#### Sense of belonging to society

Also, the sense of belonging to society of the host country has an impact on the health status and the access to health care (Bartig et al. in this journal). An operationalisation is presented elsewhere [42].

### 2.3 Indicators to capture structural factors

The aforementioned minimum and additional indicators to consider migration-related determinants should be considered in the context of structural and social determinants of health [70].

#### Education, income, occupational status – socio-economic position

Associations between the socioeconomic position of a person and (worse) health outcomes have been documented many times for sum indices [71–73]. This has also been documented many times for individual indicators, such as the level of education [74, 75], including in the two articles in this issue (Bartig et al. in this journal; Bug et al. in this journal). When considering the health situation of people with a history of migration, it is essential to use these indicators, since people with a history of migration are more often affected by educational disadvantages and poverty (risk) and more often work under precarious conditions that are less favourable to health [28].

#### Subjective social status (including social mobility)

In addition to socioeconomic position based on education, occupation, and income, subjectively perceived social status is also associated with health outcomes [37] and can be assessed using the MacArthur Scale of Subjective Social Status [42]. A subjectively perceived worse position in society can be associated with psychological stress and have a negative impact on health. For people with their own experience of migration, the hypothetical subjective social status in the country of origin can also be assessed. This concept addresses the self-assessment of what the social status would be like if the respondents had not migrated and thus reflects the respondents’ self-perceived social mobility to a certain extent. Analyses show that subjectively perceived downward mobility in the course of the migration process is related to poorer mental health [76, 77]. Operationalisation proposals are described in detail elsewhere [42].

#### Working conditions

In the area of working conditions, a number of aspects, such as precarious employment, temporary and shift work, (chain) fixed-term contracts, work in the low-wage sector, or in so-called system-relevant activities, are of importance [28, 78]. These can be associated with more difficult planning of income, residence rights, and life in general, causing psychological stress and resulting in a low socio-economic status. Jobs in so-called systemic occupations (e.g., logistics, construction, harvesting, [public] transport, retail/food trade, care) can also have an impact on health, as most recently demonstrated by the COVID-19 pandemic, e.g., lack of home office options (physical distancing) and increased exposure risks. Physical stress in the workplace also plays a role and can contribute to long-term physical suffering. All of this needs to be taken into account when analysing the health situation of people with a history of migration, as they are more likely to work in the aforementioned sectors and under the working conditions described [28]. Physical workloads can be surveyed, e.g., by means of a battery of items that was used throughout Europe in the ad hoc module of the 2013 Labour Force Survey [79, 80]. Psychological workloads can be assessed, e.g., using the Effort-Reward-Imbalance Questionnaire [81] or the Copenhagen Psychosocial Questionnaire (COPSOQ) [82]. The Employment Precariousness Scale (EPRES) by Vives et al. could also be used to assess precarious working conditions [83].

#### Housing conditions

The housing situation is also considered one of the key social determinants that can affect health in terms of quality, affordability, location, and environment of housing, among others. Cramped living conditions, especially in shared accommodation, increase the risk of transmission of infectious diseases [21]. At the same time, inadequate housing conditions can contribute to an increased experience of stress and even mental illness if, for example, there is a lack of opportunities to withdraw [84]. Factors such as the fear of being evicted from a flat or the sharp rise in rent can also contribute to the experience of stress. In addition to the size of the flat, its condition itself can also have health effects, e.g., through the occurrence of mould. Furthermore, aspects of regional deprivation are also associated with effects on health [85]. A number of approaches exist for measuring housing quality. Examples can be found in the surveys of the Socio-Economic Panel (SOEP) of the German Institute for Economic Research (DIW) with items on living environment, size, and type of house [86]. Further examples of survey instruments in the form of comprehensive measurement scales with multiple dimensions of housing quality can be found in international research [87, 88].

### 2.4 Examples of applications

A number of scientific contributions already make use of the presented single indicators, either in combination with the category of ‘migration background’ or entirely independently. In the following paragraph, we describe some examples to underline the importance of differentiated analyses.

A contribution using data of the German Health Survey for Children and Adolescents (KiGGS wave 2, 2014–2017) shows that there is no difference in the utilisation of paediatrics’ and general practitioners’ services by migration background, but within the group of children and adolescents with a migration background, there are differences in utilisation depending on the parents’ duration of residence. A shorter duration of residence is associated with lower utilisation [89]. Another contribution using the same data showed that there is no difference in the self-assessed subjective health of children and adolescents by migration background; however, reporting experiences of discrimination was associated with reporting a worse health status [90]. One further contribution using the data of KiGGS wave 2 showed a lower prevalence of diagnoses for neuro-dermatitis within the group of children and adolescents with a ‘migration background’ for those with lower socioeconomic position and for those whose parents had a duration of residence in Germany below ten years [91]. A contribution using data of the IMIRA feasibility study ‘interview survey’ [92] had shown that a shorter duration of residence, as well as a temporary residence status, were associated with lower utilisation of general practitioners’ services [93]. Recent contributions focusing on the topic of COVID-19 used similar data analysis strategies. Results of the ‘COVID-19 vaccination rate monitoring in Germany as an immigrant society (COVIMO Fokuserhebung)’, had shown that lower immunisation rates were associated with lower German language proficiency and with self-reported experiences of discrimination in health care facilities [94]. Using data of the COVID-19 Snapshot Monitoring survey (COSMO), it was possible to show that the higher probability for reporting an infection with the Severe Acute Respiratory Syndrome Coronavirus Type 2 (SARS-CoV-2) for participants with a history of migration decreased when stepwise integrating further explanatory variables into statistical models (sociodemographic characteristics, household size, household language, and occupation in the health sector) [95]. Further examples of applications are shown within this special issue (Bartig et al. in this journal; Bug et al. in this journal).

However, it is important to consider effects of mediation and moderation between different variables in statistical models and identify possible confounder-associated effect modifications. If applicable, the choice of variables integrated into a statistical model needs to be adapted. Distortions in results, as well as misinterpretations of observed differences, can be avoided this way (for examples, see [96]).

## 3. Discussion

Based on a revision of the existing set of minimum indicators for the assessment of migration status [35], a number of indicators for the analysis of migration-related determinants, as well as structural and living environment-related factors, were presented. These can be relevant in the analysis of the health situation of people with and, to some extent, people without a history of migration. This includes the recommendation to refrain from applying the concept of ‘migration background’ and to examine the presented indicators in their interactions with other social determinants of health, as well as structural aspects and living conditions. Here, a good scientific practice requires reflection on prior assumptions and a differentiated development of the research question and analysis, while taking into account relevant explanatory factors. The described indicators should be considered as possible explanatory variables. Furthermore, potential mediation and moderation effects within the observed differences in health outcomes should be addressed instead of solely focusing on ‘migration’.

It is crucial to reflect on which differences and exclusions are (re-)produced by the researcher’s own analyses and what the resulting statements can mean for the (groups of) people being studied [97]. In this context, the ethical principle of non-harm must always be respected, i.e., research should not cause harm to those being researched. Likewise, the risk of data misappropriation, i.e., the research being necessarily tied to its purpose, should be taken into account [8]. This means that the risk that data can be taken out of the research context and misused for other purposes, such as false and stigmatising representations of entire population groups, should be reflected and minimised through differentiated analyses; however, these recommendations represent only one of many necessary steps towards diversity-oriented public health research. The diversity of the population requires constant reflection on the categories and concepts applied and an ongoing process of adjustment. Therefore, some current aspects of the discourse will be taken up in the following and presented as a methodological outlook.

**Info box 2**

**Terms**

**Migration and racism**

We speak of research on migration and racism because these two aspects inevitably belong together in scientific perspectives. In addition to everyday experiences of discrimination, racism can manifest itself in the form of structural and institutional exclusion (housing, work, education, health, etc.) and power relations. The corresponding social determinants of health are relevant for a number of population groups; at the same time, not all of them have their own or a family history of migration, but they can still have experiences of othering, ethnicisation, and racism.

**Ethnicisation (also ‘culturalisation’ or ‘cultural essentialisation’)**

Ethnicisation describes social processes of assigning identities and presumed ethnic groups or ethnicities, attributing characteristics on the basis of this assignment, and of reducing differences between categories or groups of people, but also of social conflicts to ethnic or cultural characteristics. There may be self-ethnicisation and external ethnicisation (see [120], own translation).

https://www.kulturglossar.de/html/e-begriffe.html#ethnisierung

**Racialisation**

Racialisation refers to a process of biologistic and racist knowledge production. In this process, people and groups of people are assigned to ‘races’ through categorisation, homogenisation, and hierarchisation on the basis of (assumed) characteristics, such as appearance, skin colour, language, or behaviour. In this context, it is important to keep in mind that there is no such thing as a human race. Rather, it is a social reality in which *white* represents the (unmarked) norm, while non-*white* persons are racially marked (racialised) (see [121], own translation).

https://www.idaev.de/recherchetools/glossar

***White/Whiteness***

*White* does not mean the shade of a person’s skin but a socio-political norm and position of power. Therefore, *white* is often written in lower case and italics in scientific texts. *Whiteness* refers to the privileged and dominant position within social power relations in relation to racism that is kept unmarked and unnamed (see [121], own translation).

https://www.idaev.de/recherchetools/glossar

### 3.1 ‘Race’ and ethnicity: application in the German-speaking context?

The indicators described to capture migration-related determinants, as well as structural and living environment-related factors, can be measured comparatively well in health and social science surveys. In addition, there are aspects that are rather difficult to operationalise and require a thorough reflection on their utilisation. For international migration-related public health research, for example, it is relevant how ‘race’ or ethnic belonging, as well as ethnicity, can be operationalised and for which research questions and intervention strategies their operationalisation is needed. The multitude of these terms, which in part address similar and completely different dimensions, already indicates some difficulties that can be encountered when operationalising these categories in quantitative surveys [98, 99]. In Germany, a skewed data situation and deficits in representation for specific population groups have been identified in population-based epidemiological surveys, for example for ethnicised or racialised people (cf. Info box 2) or for gender differentiations [98]. For further development of health surveys, the following question must be asked: ‘how to appropriately differentiate according to the social reality’ [100, p. 1]. Furthermore, it must be taken into account that research categories and subgrouping in statistical analyses produce differences in the first place and can contribute to the reproduction of social hierarchisation and inequality [101, 102]. It is therefore important to avoid such statistical ethnification, which means that groupings or aspects of health inequality should not be reduced to factors such as origin or presumed ethnicity.

From a critical scientific perspective on racism, it has been stated that although contemporary racism is able to do without the concept of race, it often works with culturalist and ethnicising explanatory approaches and categories (see Info box 2) [103]. It assumes cultures and their supposed carriers as fixed, invariable entities. It implies differences within them that may lead to social conflicts [103–105]. Thus, ethnicity, origin, culture, or tradition are often spoken of as individual or group-related characteristics, without taking into account the legal and structural disadvantages, belonging, and discrimination [104, 106]. In this way, relevant explanatory mechanisms of health inequality are obscured and the ‘otherness’ of the supposed holders of these characteristics is emphasised and essentialised, i.e., substantiated as inherent.

A subjective assessment of self-attributed and externally attributed ethnicity(-ies) is one of the advancing concepts in current migration- and racism-related quantitative research that is currently also being discussed in detail in surveys in Germany [98, 102]. Within the framework of conceptual preparations for the survey study ‘German Health Update: Fokus (GEDA Fokus)’ among people with selected citizenships [107], a survey of the subjective assessment of self-attributed and other-attributed ethnicity(-ies) was also tested at the RKI as part of cognitive pre-tests [108]. The results showed that, especially for quantitative surveys, the list of possible answer categories, which must include open-ended text due to the nature of the research question, can become quite long. In particular, the processing of open information from multilingual questionnaires presents a challenge. The question of analysis options also needs to be discussed. Also, experiences of everyday and structural discrimination cannot be depicted in this survey approach.

In surveys that focus on specific groups of people with a history of migration, instruments for self-attribution and external attribution (subjective self-disclosure/self-identification or self-perceived external ascription) are already being used [109]. The development of a feasible procedure for population-based studies involving communities and those affected is currently still pending for the German-speaking context [98].

### 3.2 Intersectionality: a necessary perspective

An intersectional perspective is desirable for future research on migration and health. The term intersectionality describes the interweaving of different structural categories that generate inequality. Originally, the concept was developed by Black activists in the United States of America to conceptualise the multiple oppression of Black women, as it could not be captured by existing methodological approaches to the analysis of race or gender as single axes [110–114]. Since then, intersectionality has established itself as a scientific concept and has in many ways been detached from its original foundations [115]. The concept of intersectionality helps to address formations of complex social inequality and describe categories such as gender, age, migration-related determinants (e.g., country of birth, citizenship), ethnicity (or experiences of ethnicisation and racist discrimination), class, sexual identity, and orientation in their interconnections and interactions [114, 116]. There can be multiple discriminations and at the same time, very specific life experiences and situated knowledge emerge from the entanglement of social categories, i.e., knowledge that is never neutral but always shaped by specific and subjective experiences, localisations, and perspectives. From an intersectional perspective, it is necessary to consider multiple and intertwined dimensions of discrimination instead of focusing on single categories, such as racial discrimination [117]. The proposed instrument for measuring subjectively perceived discrimination allows for the specification of multiple dimensions of discrimination from the perspective of those affected [42].

The theoretical concept of intersectionality is also gaining importance in the field of health sciences. Methodological approaches for using the concept in quantitative studies are being discussed and developed [118]. Questions of applicability and ‘analytical sensitivity’ [119, p. 795] in quantitative studies still need to be thoroughly explored. The future integration of the concept should be done with the clear aim of making social disadvantages and aspects of health inequalities empirically visible. We advocate for further development of these approaches in public health research and an analysis of the health-relevant structural and social determinants that is as differentiated as possible.

## 4. Conclusion

Public health and epidemiological research, especially in the field of migration and racism, operate in an area of tension: on the one hand, there is the objective of a statistical representation of different population groups in order to ideally generate ‘equality data for equal participation’ [122] and to make health inequalities and the underlying mechanisms visible. On the other hand, this entails the risk of external attributions, discrimination, and misinterpretations. In principle, however, the ethical principle of non-harm applies at all times [8]. In the course of our preliminary work and discussions and also in this article, we have tried to approach this topic and explore the possibilities and necessities of reflective, responsible, and discrimination-sensitive research – research that at the same time enables state-of-the-art analyses across different contexts and under specific operational circumstances of the respective research setting and can provide a basis for the targeted derivation of recommendations for diversity-friendly health care and policy.

## Key statements

- The statistical category ‘migration background’ is, for methodological and content-related reasons, not useful for differentiated analyses in health science.
- The country of birth of the respondents and their parents, citizenship(s), year of immigration, current residence status, and self-assessed proficiency of the German language should be collected as minimum indicators.
- The minimum indicators should not be summed up into one category such as ‘migration background’ or other index variables.
- Depending on the research question, the minimum indicators should be supplemented by additional indicators, such as self-reported discrimination, reasons for migration, social support, sense of belonging to society, or subjective social status.
- In addition to migration-related factors, analysis should also consider further explanatory factors, such as socio-economic position or working and housing conditions.

## Figures and Tables

**Table 1.** Categories and definitions concerning migration in official statistics and in the KiGGS study Source: own table

Category	Definition	Share of the population according to microcensus data 2020 [3]
**Official statistics**		
People with migration background	‘Overall, the population with migration background is comprised of all people who themselves or at least one of their parents was born with a citizenship other than German. (…) This includes foreigners, (late) repatriates, naturalised people, and people who received German citizenship via adaptation, as well as the children born with German citizenship of these four groups.’ [4]*	27% (total population) 24% (adult population) 39% (population below 18 years of age)
Foreigners	‘Foreigners are persons who are not Germans in accordance with article 116 paragraph 1 of the Basic Constitutional Law. This includes stateless persons, as well as persons with unexplained citizenship. (…) They can either be born in Germany or immigrated.’ [4]*	12% (total population) 13% (adult population) 12% (population below 18 years of age)
People with own experience of migration	‘A person has their own experience of migration when s/he was born abroad. S/he is an immigrant.’ [4]*	17% (total population) 18% (adult population) 9% (population below 18 years of age)
**Definition according to Robert Koch Institute, German Health Survey for Children and Adolescents (KiGGS)**		
Children and adolescents with migration background	‘Children who immigrated themselves from another country and who have at least one parent who was not born in Germany; or children whose both parents had immigrated or whose both parents do not have German citizenship.’ [5, p. 11]^*^ ‘In the few cases where information on the country of birth of the child or on the birth countries/citizenship of the parents were lacking, the language spoken at home was used as an indicator for the migration background.’ [6, p. 14]^*^	

^*^represents our own translation

**Table 2.** Indicators for data collection on migration-related determinants, as well as relevant structural factors Source: own table

Indicators for the collection of migration-related determinants
**Minimum indicators**
► country of birth ► country of birth of parents ► citizenship(s) ► year of immigration ► residence status ► German language proficiency
**Social and migration-related additional indicators**
► reasons for migration ► self-reported discrimination ► social support ► sense of belonging to society
**Indicators for the collection of data on structural factors**
► education, income, occupational position (socioeconomic position) ► subjective social status (including social mobility) ► working conditions ► housing conditions
